# Apolipoprotein A-I gene transfer exerts immunomodulatory effects and reduces vascular inflammation and fibrosis in ob/ob mice

**DOI:** 10.1186/s12950-016-0131-6

**Published:** 2016-08-02

**Authors:** Frank Spillmann, Bart De Geest, Ilayaraja Muthuramu, Ruhul Amin, Kapka Miteva, Burkert Pieske, Carsten Tschöpe, Sophie Van Linthout

**Affiliations:** 1 Department of Cardiology, Charité-University-Medicine Berlin, Campus Virchow Klinikum (CVK), Berlin, Germany; 2 Catholic University of Leuven, Center for Molecular and Vascular Biology, Department of Cardiovascular Sciences, Leuven, Belgium; 3 Berlin-Brandenburg Center for Regenerative Therapy (BCRT), Charité-University-Medicine Berlin, Campus Virchow Klinikum (CVK), Südstrasse 2, 13353 Berlin, Germany; 4 Deutsches Zentrum für Herz Kreislaufforschung (DZHK), Standort Berlin/Charité, Berlin, Germany; 5 Department of Cardiology, Deutsches Herzzentrum Berlin (DHZB), Berlin, Germany

**Keywords:** HDL, Immunomodulation, Aorta, Vascular fibrosis

## Abstract

**Background:**

Obesity is associated with vascular inflammation, fibrosis and reduced high-density lipoproteins (HDL)-cholesterol. We aimed to investigate whether adenoviral gene transfer with human apolipoprotein (apo) A-I (*Ad.A-I*), the main apo of HDL, could exert immunomodulatory effects and counteract vascular inflammation and fibrosis in ob/ob mice.

**Methods:**

*Ad.A-I* transfer was performed in 8 weeks (w) old ob/ob mice, which were sacrificed 7 w later. The aorta was excised for mRNA analysis and the spleen for splenocyte isolation for subsequent flow cytometry and co-culture with murine fibroblasts. HDL was added to mononuclear cells (MNC) and fibroblasts to assess their impact on adhesion capacity and collagen deposition, respectively.

**Results:**

*Ad.A-I* led to a 1.8-fold (*p <* 0.05) increase in HDL-cholesterol versus control ob/ob mice at the day of sacrifice, which was paralleled by a decrease in aortic TNF-α and VCAM-1 mRNA expression. Pre-culture of MNC with HDL decreased their adhesion to TNF-α-activated HAEC. *Ad.A-I* exerted immunomodulatory effects as evidenced by a downregulation of aortic NOD2 and NLRP3 mRNA expression and by a 12 %, 6.9 %, and 15 % decrease of the induced proliferation/activity of total splenic MNC, CD4+, and CD8+ cells in ob/ob *Ad.A-I* versus control ob/ob mice, respectively (*p <* 0.05). *Ad.A-I* further reduced aortic collagen I and III mRNA expression by 62 % and 66 %, respectively (*p <* 0.0005), and abrogated the potential of ob/ob splenocytes to induce the collagen content in murine fibroblasts upon co-culture. Finally, HDL decreased the TGF-ß1-induced collagen deposition of murine fibroblasts *in vitro*.

**Conclusions:**

Apo A-I transfer counteracts vascular inflammation and fibrosis in ob/ob mice.

**Electronic supplementary material:**

The online version of this article (doi:10.1186/s12950-016-0131-6) contains supplementary material, which is available to authorized users.

## Background

Obesity is an inflammatory disorder [1] associated with endothelial dysfunction [2], vascular fibrosis [3], and an increased cardiovascular risk [4, 5]. It is characterized by a chronic low-grade activation of the innate immune system [1]. This involves among others the NOD-like receptor (NLR) family of pattern recognition receptors, including the NLRP3 inflammasome [6, 7], which senses obesity-induced stress signals like cholesterol crystals [6], and NOD2 [8]. Both NLRP3 and NOD2 are expressed on endothelial cells, stimulate endothelial inflammation [8–10], and are involved in the pathogenesis of fibrosis [11, 12]. Inflammation is a well-established trigger of fibrosis [13]: it can “classically” induce the activation/proliferation and transdifferentiation of resident fibroblasts to myofibroblasts and induce endothelial-to-mesenchymal-transition (EndMT) [14], a process by which endothelial cells transdifferentiate into mesenchymal-like cells, characterized by induced α-smooth muscle actin expression, loss of endothelial cell markers, and increased collagen deposition [15–17].

Obesity is associated with low high-density lipoprotein (HDL) cholesterol levels, by which the body weight inversely correlates with HDL cholesterol [18]. HDL cholesterol levels are also decreased in several other inflammatory disorders including atherosclerosis [19], systemic lupus erythematosus [20], and rheumatoid arthritis [21], suggesting a link between HDL and the immune response. Apolipoprotein A-I (apo A-I) is the principal apolipoprotein of HDL and plasma levels of this apolipoprotein strongly correlate with HDL cholesterol levels. At the molecular and cellular level, HDL/apo A-I are known to reduce Toll like receptor 4 expression [22], inhibit antigen presentation [23], and reduce T cell proliferation [24]. Besides these immunomodulatory characteristics, HDL/apo A-I are particularly known for their endothelial-protective properties including their capacity to facilitate vascular relaxation via activation of eNOS [25], to restore impaired NO bioavailability [26], and to decrease the expression of vascular adhesion molecules [27]. Besides these well-established endothelial-protective effects, there is accumulating evidence that HDL and apo A-I also have anti-fibrotic properties: overexpression of apo A-I in the lung abrogates fibrosis in experimental silicosis [28], low apo A-I levels predict liver fibrosis in hepatitis C patients [29], and HDL inversely correlate to the serum marker of cardiac fibrosis, procollagen type III aminopeptide [30]. Furthermore, we demonstrated that human *apo A-I* gene transfer decreased cardiac fibrosis in an experimental rat model of diabetic cardiomyopathy [27]. With respect to the aorta, it has been shown that infusions of apo A-I mimetic peptide reduce fibrosis in the aortic root in a mouse model of aortic valve stenosis [31]. Recently, we showed that HDL supplementation to human aortic endothelial cells (HAEC) decreased transforming growth factor (TGF)-ß1-induced EndMT [14].

Given the endothelial-protective, anti-fibrotic, and immunomodulatory effects of HDL/apo A-I on the one hand, and the occurrence of vascular inflammation and fibrosis, and innate immune activation under obese conditions on the other hand, we aimed to investigate whether human *apo A-I* gene transfer can decrease aortic inflammation and fibrosis in leptin-deficient ob/ob mice, which are overweight [32], insulin-resistant [33], and develop vascular inflammation and fibrosis [34, 35]. We next aimed to understand whether apo A-I/HDL might also affect vascular inflammation and fibrosis via systemic immunomodulation. In this respect, *in vitro* studies were conducted to get insights in whether (i) HDL modulate the binding capacity of MNC to TNF-α-activated HAEC; and whether (ii) modulation of splenic activity following apo A-I transfer influences collagen production upon co-culture of those splenocytes with fibroblasts.

## Methods

### Animals and study design

Male B6.V-Lepod/JRj mice (ob/ob; Janvier Labs, Le Genest-Saint-Isle, France) were intravenously injected via the tail vein with saline or with 5 x 10^10^ total particles of the E1E3E4-deleted adenoviral vector *Ad.A-I* expressing human apo A-I [36] or of *Ad.Null* containing no expression cassette [36], at the age of 8 weeks. As control reference, age-matched male C57BL/6 mice were injected with saline. Mice were sacrificed 7 weeks later, the aorta was excised and snap-frozen in liquid nitrogen for molecular biology and the spleen was isolated for splenocyte preparation and subsequent flow cytometry or co-culture with murine fibroblasts. Blood was withdrawn from the retro-orbital plexus at day 7, 21, 35, and 49 after gene transfer for determination of human apo A-I concentrations. At day 45 after gene transfer or saline injection, an intraperitoneal glucose tolerance test was performed. The investigation conforms with the *Guide for the Care and Use of Laboratory Animals* published by the US National Institutes of Health (NIH Publication No. 85–23, revised 1996) and was approved by the Institutional Animal Care and Research Advisory Committee of the Catholic University of Leuven (Approval number: P154/2013).

### Human apo A-I ELISA

Human apo A-I levels were determined by sandwich ELISA as described previously [37].

### Plasma lipid analysis

Mouse lipoproteins were separated by density gradient ultracentrifugation as described by Jacobs *et al*. [38]. Fractions were stored at −20 °C until analysis. Cholesterol in lipoprotein fractions was determined with commercially available enzymes (Roche Diagnostics, Basel, Switzerland). Precipath L (Roche Diagnostics) was used as a standard.

### Glucose tolerance test

Glucose tolerance test was performed by intraperitoneal injection of glucose (2 g/kg) after 6 h (h) of fasting as described by Hofmann *et al*. [39]. Tail blood glucose levels were measured with an Accu-Chek® Active Glucometer (Roche Applied Science, Penzberg, Germany) before (0 min) and at 15, 30, 60 and 120 min after injection.

### Splenocyte isolation

Splenocytes were isolated from the different experimental groups as described previously [40].

### (Splenocyte) mononuclear cell, CD4-, and CD8-T cell proliferation

Splenocytes were labeled with 10 μM of succinimidyl ester of carboxyfluorescein diacetate (CFSE Cell Tract™; Invitrogen, Carlbard, CA, USA) to be able to measure cell proliferation, indicative for MNC activation, as described previously [41]. Therefore, splenocytes were cultured in RPMI1640 medium (Invitrogen, Heidelberg, Germany), supplemented with 10 % FBS and 1 % penicillin/streptomycin for 72 h. Then, cells were stained with monoclonal anti-CD4 or anti-CD8 antibodies (BD Biosciences, Franklin Lakes, NJ, USA), followed by flow cytometry on a MACSQuant Analyzer (Miltenyi Biotec, Bergisch Gladbach, Germany) and analysis with FlowJo 8.7. software (Tree Star) for the calculation of the division index, i.e. the average number of cell divisions that the responding cells undergo (i.e., ignores peak 0).

### TGF-ß1 expressing mononuclear cells

For the analysis of MNC expressing TGF-ß1, splenocytes were intracellulary stained with anti-TGF-ß1 (clone TW7-16B4 recognizing latency associated peptide (LAP), latent TGF-ß, and pro-TGF-ß; Biolegend, San Diego, CA, USA) after fixation. Splenocyte samples were acquired on a MACSQuant Analyzer (Miltenyi Biotec, Bergisch Gladbach, Germany). Analysis of flow cytometry data was performed using FlowJo software version 8.8.6. (Tree Star Inc.).

### Cell culture

Murine C4 fibroblasts, a murine fibroblast cell line derived from embryonic BALB/c mice by SV40 infection *in vitro* [42], were cultured in Basal Iscove medium supplemented with 10 % FBS and 1 % Penicillin/Streptomycin. Twenty-four h after plating at a cell density of 10,000 cells/96-well, cells were stimulated with/out 10 ng/ml TGF-ß1 (R&D Systems, Minneapolis, USA) under serum starvation conditions, i.e. Basal Iscove medium with 0.01 % FBS and 1 % Pencillin/Streptomycin, in the presence or absence of 50 μg/ml of HDL (MP Biomedicals, Solon, Ohio, USA) for 24 h. Next, cells were fixed in methanol overnight at −20 °C for subsequent collagen deposition staining.

Human aortic endothelial cells (HAEC; Lonza, Basel, Switzerland) were cultured in Endothelial Basal Medium (Lonza) supplemented with the EGM-2 BulletKit (Lonza) on pre-coated (coating solution containing 0.02 % gelatin from bovine skin type B and 125 ng/ml fibronectin from bovine plasma; both Merck Chemicals, Darmstadt; Germany) cell culture flasks.

### Co-culture of fibroblasts with splenocytes

Murine C4 fibroblasts were plated at a cell density of 10,000 cells/96-well in Basal Iscove medium supplemented with 10 % FBS and 1 % Penicillin/Streptomycin. Twenty-four h after plating, medium was removed and splenocytes isolated from control, ob/ob, ob/ob *Ad.Null*, and ob/ob *Ad.A-I* mice were added to the fibroblasts at a ratio of 10 splenic cells to 1 fibroblast in RPMI1640 medium (Invitrogen, Heidelberg, Germany) 10 % FBS, 1 % Penicillin/Streptomycin. After 24 h, splenocytes were removed and the murine fibroblasts were fixed in methanol overnight at −20 °C for subsequent collagen deposition staining.

### Quantification of collagen deposition

Collagen deposition was determined as described previously [14, 43]. In brief*,* after overnight fixation in methanol at −20 °C, cells were washed once with PBS and incubated in 0.1 % Direct Red 80 (Sirius red; Merck Chemicals, Darmstadt; Germany) staining solution at RT for 60 min. After second washing with PBS, the Sirius red staining of the murine fibroblasts was eluted in 0.1 N sodium hydroxide at RT for 60 min on a rocking platform. The optical density, representative for the accumulation of collagen, was measured at 540 nm. For normalization to cell amount, murine fibroblasts were stained with crystal violet (Merck Chemicals, Darmstadt, Germany) and the absorbance was measured at 495 nm. Data are represented as the ratio of the absorbance at 540 nm (Sirius Red) towards the absorbance at 495 nm (crystal violet).

### Human peripheral blood mononuclear cell isolation

Blood was withdrawn from healthy donors (aged 40 to 65) with approval (EA4/115/11) from the Ethical Commission (Charité, CBF, Berlin). Human peripheral blood mononuclear cells (MNC) were isolated from the blood samples by density-gradient centrifugation (Biocoll; Merck Millipore, Darmstadt, Germany). Cells were stored in liquid nitrogen until use.

### Adhesion assay

HAEC were plated in gelatin/fibronectin-coated black solid bottom 96-well plates, cultured for 24 h and treated with 10 ng/ml TNF-α (BD Pharmingen, Franklin Lakes, NJ, USA) in Endothelial Basal Medium for 4 h prior to addition of MNC. DiO-labeled human MNC (DiO, Molecular Probes, Life Technologies) were cultured with/out 50 μg/ml of HDL for 24 h in 24-well plates and next added to TNF-α-stimulated HAEC at a ratio of 1:10 for 30 min. Subsequently, MNC were removed by aspiration and HAEC were washed once with PBS. PBS was added to each well and DiO-fluorescence was detected with a Mithras LB 940 multitechnology microplate reader (Berthold technologies) at wavelengths of 485 nm for excitation and 535 nm for emission and corresponding software (MikroWin 2000, Mikrotek Laborsysteme GmbH).

### Gene expression analysis

RNA from the aorta was isolated using the RNeasy Mini Kit according to the manufacturer’s protocol (Qiagen GmbH, Hilden, Germany), followed by cDNA synthesis. To assess the mRNA expression of the target genes TNF-α, VCAM-1, CD4, CD8, NOD2, NLRP3, collagen I and III, real-time PCR (Eppendorf Mastercycler epgradient realplex, Hamburg, Germany) was performed using gene expression assays for TNF-α Mm00443258_m1, VCAM-1 Mm01320970_m1, CD4 Mm00442754_m1, and CD8a Mm01182107_g1, nucleotide-binding oligomerization domain containing (NOD) 2 Mm00467543_m1, nucleotide-binding domain, leucine-rich-containing family, pyrin domain-containing (NLRP) 3 Mm00840904_m1, Col1a1 Hs00164004_m1, and Col3a1 Hs00943809_m1 from Applied Biosystems, respectively. mRNA expression was normalized to the housekeeping gene CDKN1b (gene expression assay Hs00153277_m1) and relatively expressed with the control group set as 1.

### Statistical analysis

Data are presented as mean ± SEM. Statistical differences between groups were assessed with Ordinary One-way ANOVA. Differences were considered to be significant when the P-value was lower than 0.05.

## Results

### Metabolic modulation after human apo A-I transfer in ob/ob mice

**Fig. 1:**
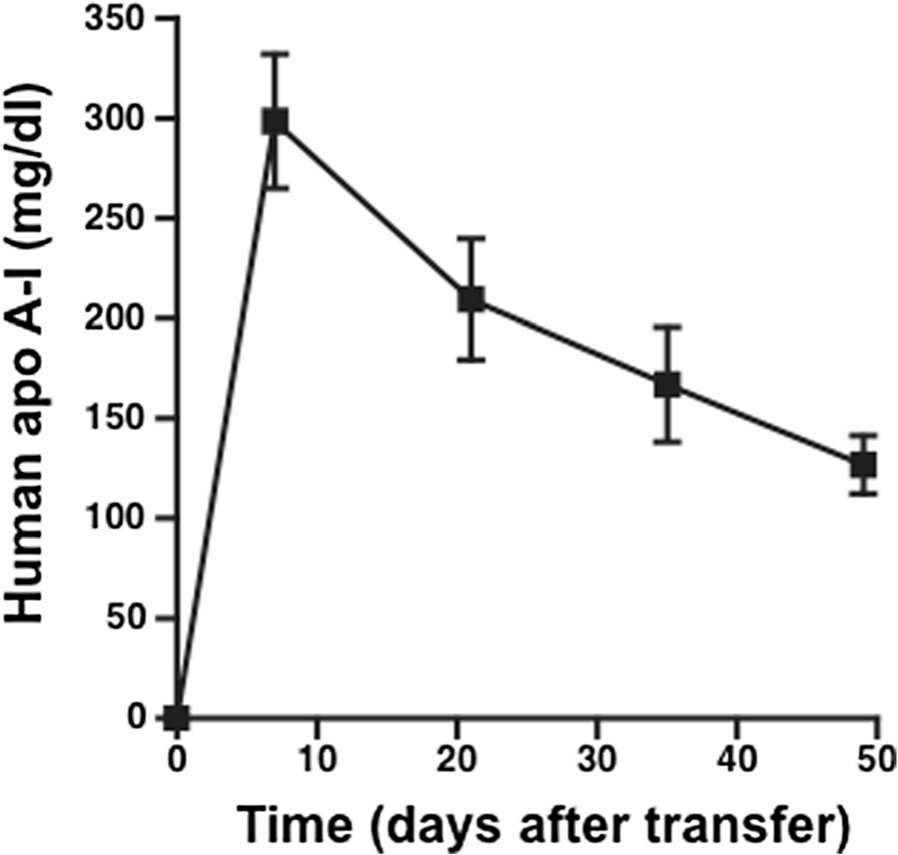
Human apo A-I levels after *apo A-I* gene transfer in ob/ob mice. Data represent mean ± SEM of *n =* 4 mice

Human *apo A-I* gene transfer resulted in persistent expression of human apo A-I in ob/ob mice (Fig. 1) leading to a 79 % (*p <* 0.001) and 68 % (*p <* 0.001) increase in HDL cholesterol levels at the day of sacrifice compared to ob/ob and ob/ob *Ad.Null* mice, respectively (Table 1). At this age, ob/ob mice were still normoglycemic (Additional file 1: Figure S1A), but already glucose intolerant as evidenced by an intraperitoneal glucose tolerance test (Additional file 1: Figure S1B). *Ad.A-I* transfer did not improve glucose intolerance (Additional file 1: Figure S1B).

**Table 1:** Plasma cholesterol, non-HDL cholesterol, HDL cholesterol and plasma triglycerides at 6 weeks after saline injection or gene transfer

	Cholesterol	Non-HDL Cholesterol	HDL Cholesterol	Triglycerides
C57BL/6	66.7 ± 7.3	15.4 ± 2.0	51.3 ± 5.9	45.6 ± 4.4
Ob/ob	144 ± 7^§§§^	71.8 ± 3.9^§§§^	72.7 ± 4.3^§^	51.6 ± 3.9
Ob/ob *Ad.Null*	153 ± 2^§§§^	76.0 ± 2.5^§§§^	77.5 ± 2.7^§§^	61.3 ± 5.1
Ob/ob *Ad.A-I*	218 ± 8^§§§$$$***^	87.1 ± 4.3^§§§$^	130 ± 5^§§§$$$***^	96.3 ± 11.2^§§§$$$**^

All data are expressed in mg/dl and represent means + SEM (*n =* 7 for C57BL/6, *n =* 11 for ob/ob, *n =* 12 for ob/ob *Ad.Null*, *n =* 12 for *ob/ob Ad.A-I*). §: *p <* 0.05; §§: *p <* 0.01; §§§: *p <* 0.001 versus C57BL/6. $: *p <* 0.05; $$$: *p <* 0.001 versus ob/ob. **: *p <* 0.01; ***: *p <* 0.001 versus ob/ob *Ad.Null*

### Human apo A-I transfer exerts anti-inflammatory and immunomodulatory effects in ob/ob mice

**Fig. 2:**
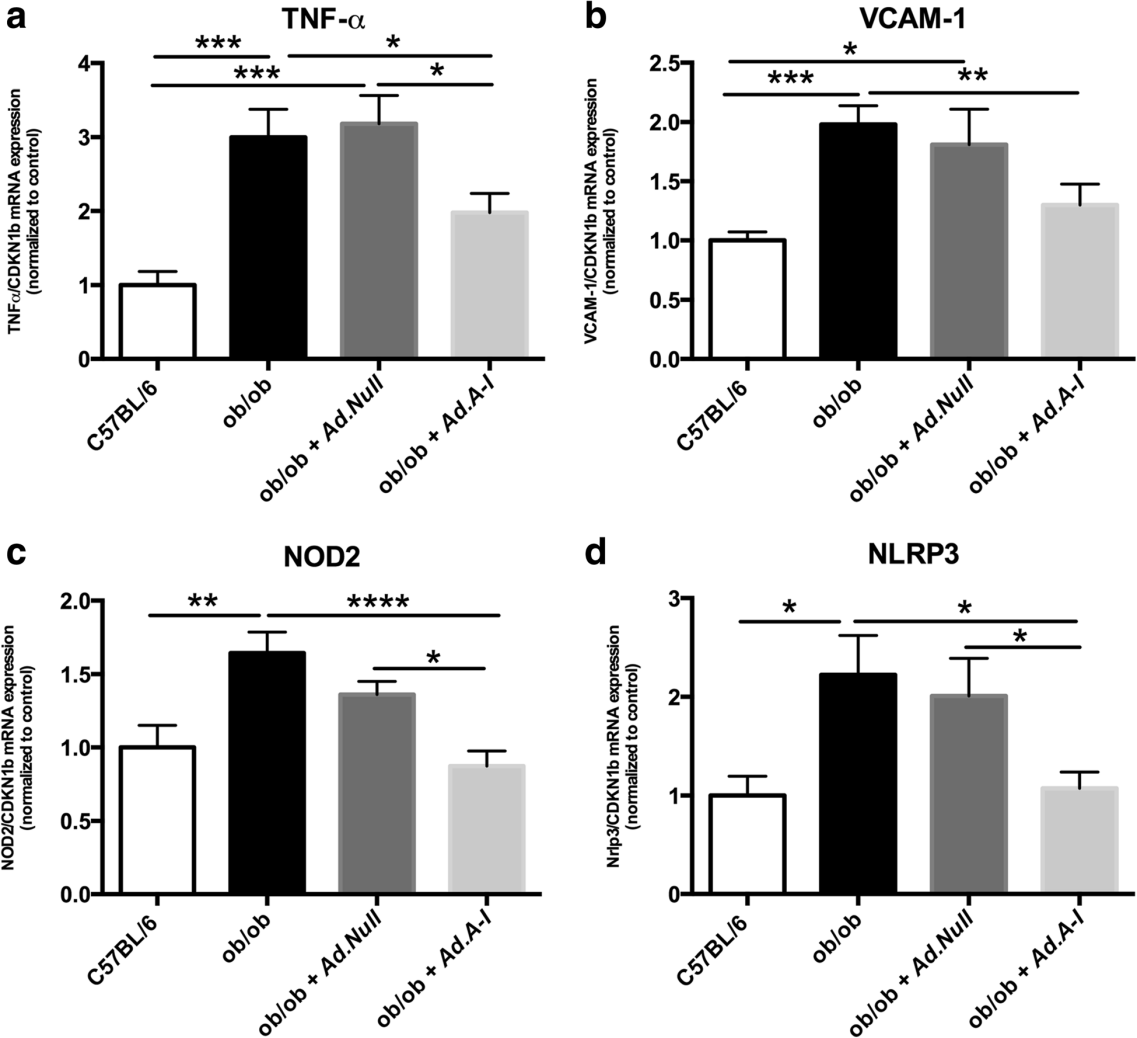
Impact of *apo A-I* gene transfer on aortic TNF-α, VCAM-1, NOD2, and NLRP3 mRNA expression in ob/ob mice. Bar graphs represent the mean ± SEM of aortic **a.** TNF-α, **b.** VCAM-1, **c.** NOD2 and **d**. NLRP3 mRNA expression with *n =* 6-7 for C57BL/6, *n =* 11 for ob/ob, *n =* 8–10 for ob/ob *Null* and *n =* 10 for ob/ob *A-I*; *p < 0.05, **p < 0.01, ***p < 0.005 and ****p < 0.0001

**Fig. 3:**
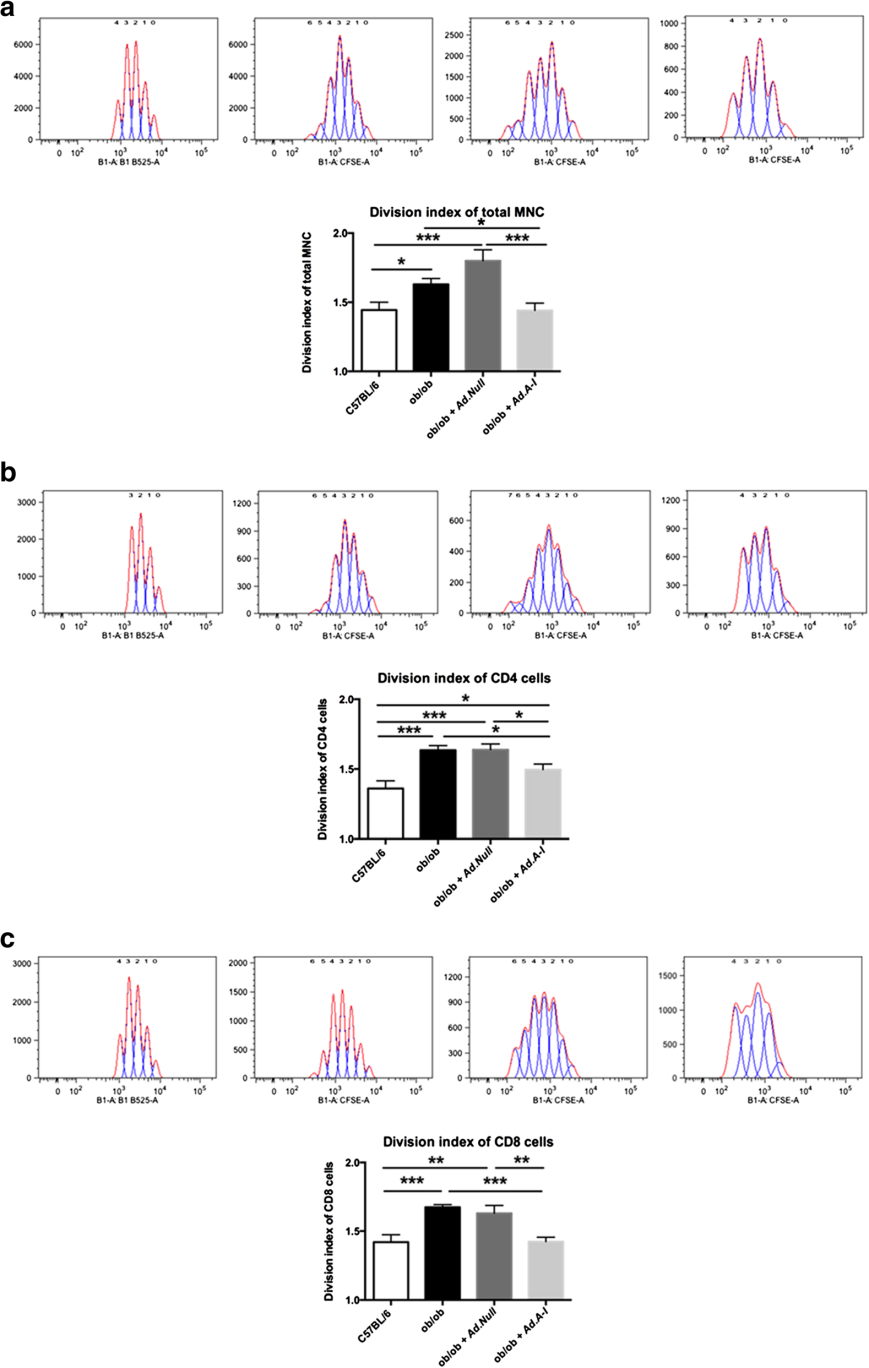
Impact of *apo A-I* gene transfer on the activity of splenic mononuclear cells, CD4+ and CD8+ T cells in ob/ob mice. Upper panels: representative peaks, indicative for the amount of cell divisions of splenic **a.** mononuclear cells (MNC), **b.** CD4+ and **c.** CD8+ T cells from C57BL/6, ob/ob, ob/ob *Ad.Null*, and ob/ob *Ad.A-I* mice, as indicated, are shown. Lower panels: Bar graphs represent the mean ± SEM of the division index of splenic **a.** MNC, **b.** CD4+ and **c.** CD8+ T cells with *n =* 5-6/group; *p < 0.05, **p < 0.005 and ***p < 0.001

**Fig. 4:**
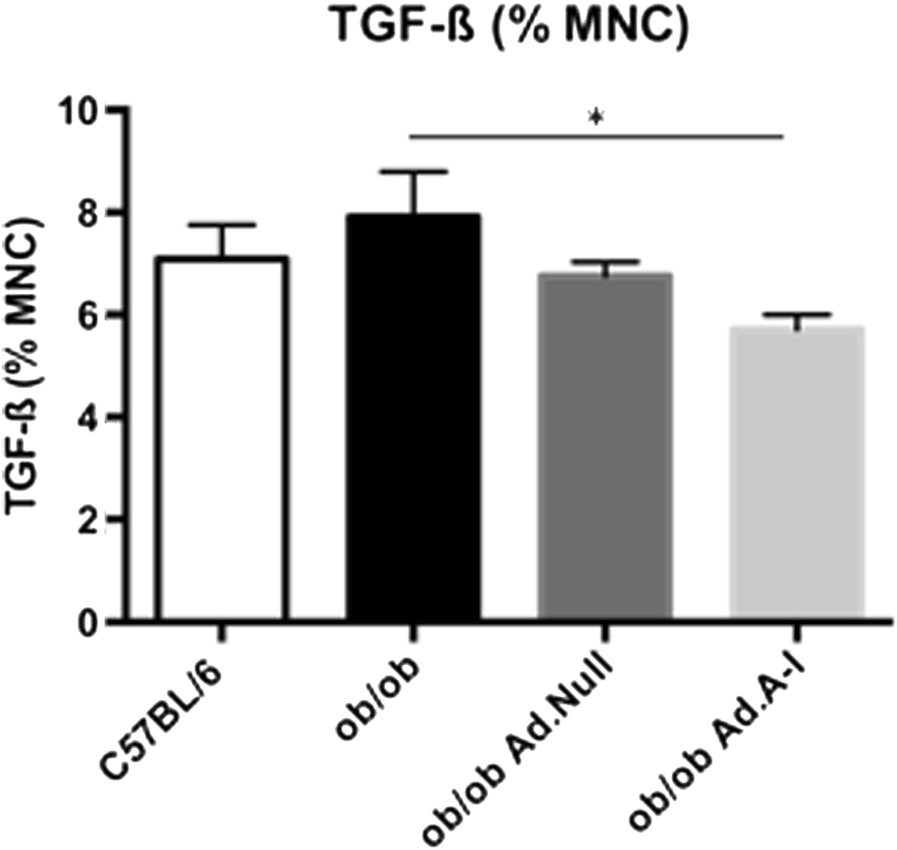
Impact of *apo A-I* gene transfer on splenic TGF-ß + mononuclear cells. Bar graphs represent the mean ± SEM of the % of splenic TGF-ß + mononuclear cells (MNC) with *n =* 5-6/group; *p < 0.05

**Fig. 5:**
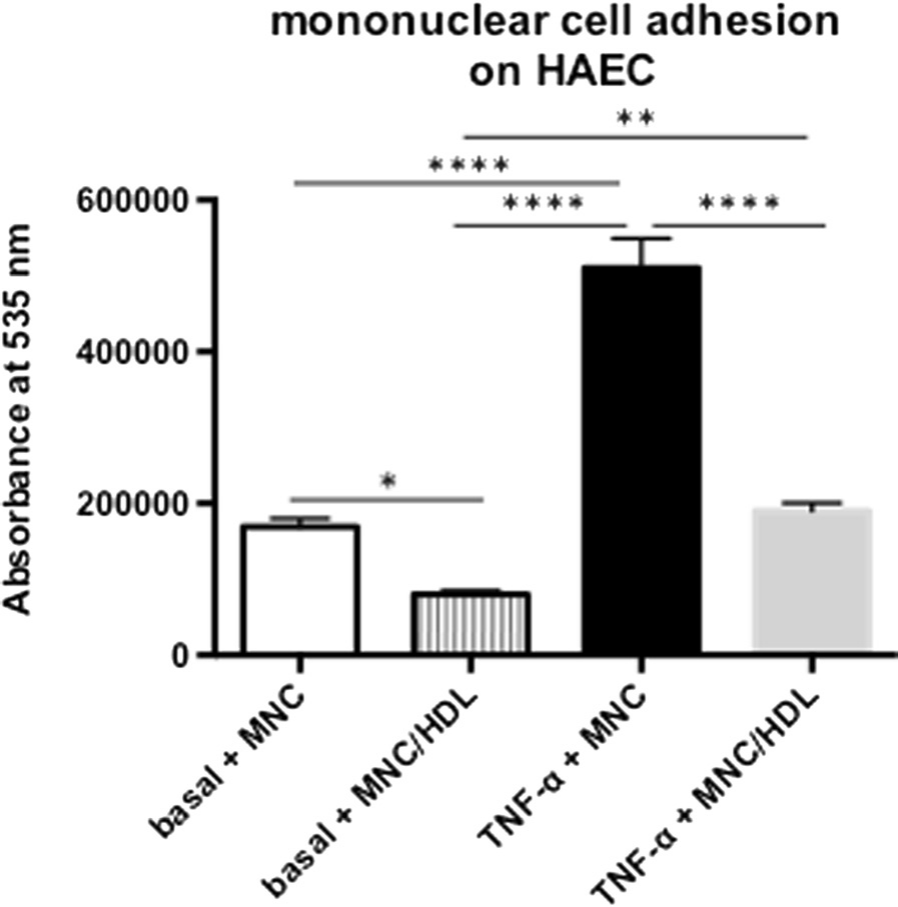
Supplementation of high-density lipoproteins on human mononuclear cells reduces their subsequent adhesion to human aortic endothelial cells. Bar graphs represent the mean ± SEM of the absorbance at 535 nm with *n =* 12/group for the untreated MNC groups and *n =* 9/group for the HDL-treated MNC groups; *p < 0.05, **p < 0.005, and ****p < 0.0001

Given the occurrence of inflammation in the vasculature under obesity on the one hand [1] and the anti-inflammatory properties of HDL/apo A-I on the other hand [27, 44], we first evaluated whether *apo A-I* gene transfer could reduce aortic inflammation in obese mice. *Apo A-I* gene transfer decreased aortic inflammation present in ob/ob mice as indicated by a 34 % (*p <* 0.05) and 34 % (*p <* 0.01) decrease in aortic TNF-α and VCAM-1 mRNA expression versus ob/ob mice, respectively (Fig. 2a-b), and a 65 % (*p =* 0.066) and 62 % (*p =* 0.076) decline in CD4 and CD8 mRNA expression versus ob/ob mice, respectively (CD4 mRNA C57BL/6: 1 ± 0.28, ob/ob: 1.9 ± 0.44, ob/ob *Ad.Null*: 2.2 ± 0.69, ob/ob *Ad.A-I*: 0.66 ± 0.14; CD8 mRNA C57BL/6: 1.0 ± 0.32, ob/ob: 2.9 ± 0.94, ob/ob *Ad.Null*: 3.3 ± 1.0, ob/ob *Ad.A-I*: 1.1 ± 0.19; with *p <* 0.05 for ob/ob *Ad.A-I* versus ob/ob *Ad.Null*). Further evaluation of the pattern recognition receptor NOD2, which can be induced by TNF-α [9], and of the pattern recognition receptor NLRP3, demonstrated a 47 % (*p <* 0.0005) and 52 % (*p <* 0.05) decline, respectively, in their mRNA expression in ob/ob *Ad.A-I* versus ob/ob mice (Fig. 2c-d). To evaluate whether these changes in vascular inflammation and innate immunity following *apo A-I* gene transfer in ob/ob mice were accompanied with systemic immunomodulation, the impact of *Ad.A-I* on the activation/proliferation state of MNC, CD4+, and CD8+ T cells and on splenic TGF-ß1-expressing MNC was determined. Ob/ob mice exhibited activated splenic MNC, CD4+, and CD8+ T cells as shown by a 13 % (*p <* 0.05), 18 % (*p <* 0.005), and 18 % (*p <* 0.005) higher proliferation capacity compared to respective splenic cells from control C57BL/6 mice (Fig. 3a-c). In contrast, *apo A-I* gene transfer decreased the activation/proliferation of splenic total MNC, CD4+, and CD8+ cells in ob/ob mice by 12 % (*p <* 0.05), 6.9 % (*p <* 0.05), and 15 % (*p <* 0.001), respectively (Fig. 3a-c), and reduced the percentage of MNC expressing TGF-ß1 by 28 % (*p <* 0.05) in ob/ob mice (Fig. 4). To further evaluate whether the immunomodulatory effects of HDL also include their ability to reduce the capacity of MNC to bind to endothelial cells, MNC pre-cultured with/out HDL were added to TNF-α-stimulated HAEC and their adhesion was measured. Pre-culture of MNC with HDL resulted in a 63 % (*p <* 0.0001) lower adhesion to TNF-α-stimulated HAEC compared to untreated MNC (Fig. 5).

### Anti-fibrotic effects after human apo A-I transfer in ob/ob mice

**Fig. 6:**
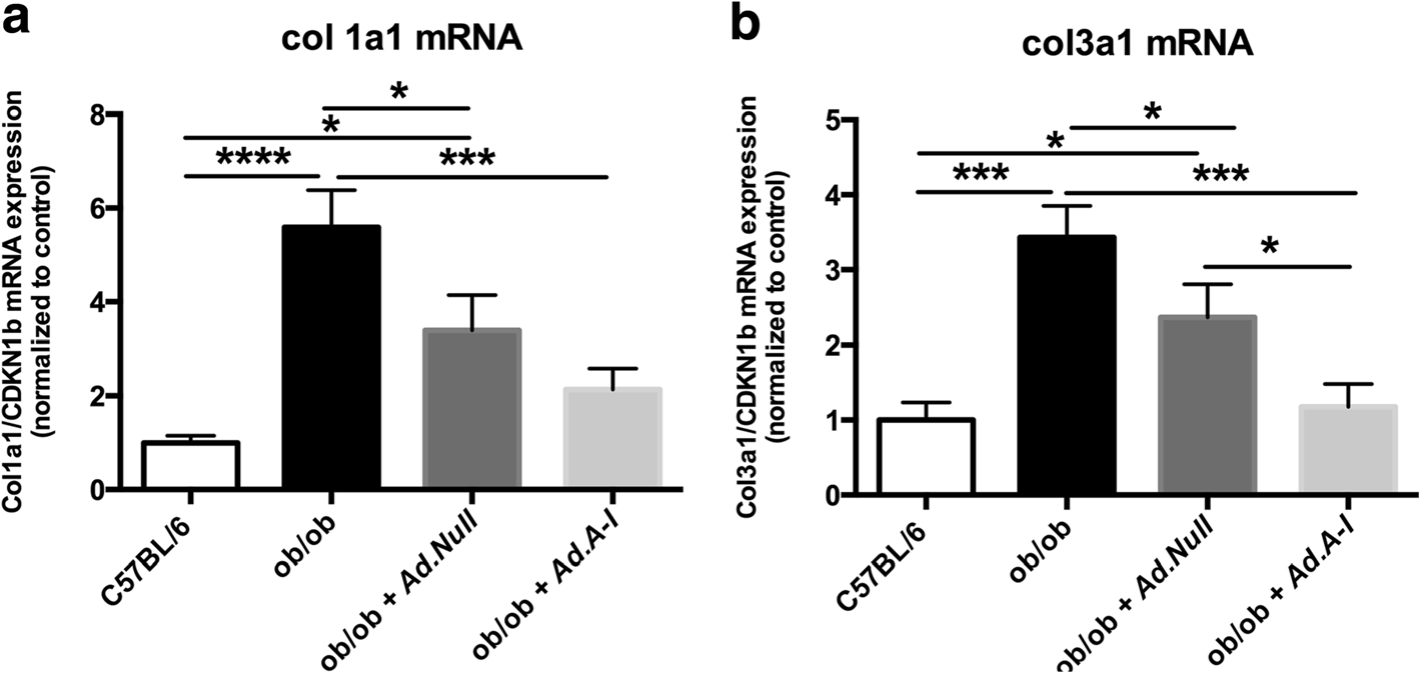
Impact of *apo A-I* gene transfer on aortic collagen I and III mRNA expression in ob/ob mice. Bar graphs represent the mean ± SEM of aortic **a**. collagen I and **b.** collagen III mRNA expression with *n =* 7 for C57BL/6, *n =* 9-10 for ob/ob groups; *p < 0.05, **p < 0.0005, and ***p < 0.0001

**Fig. 7:**
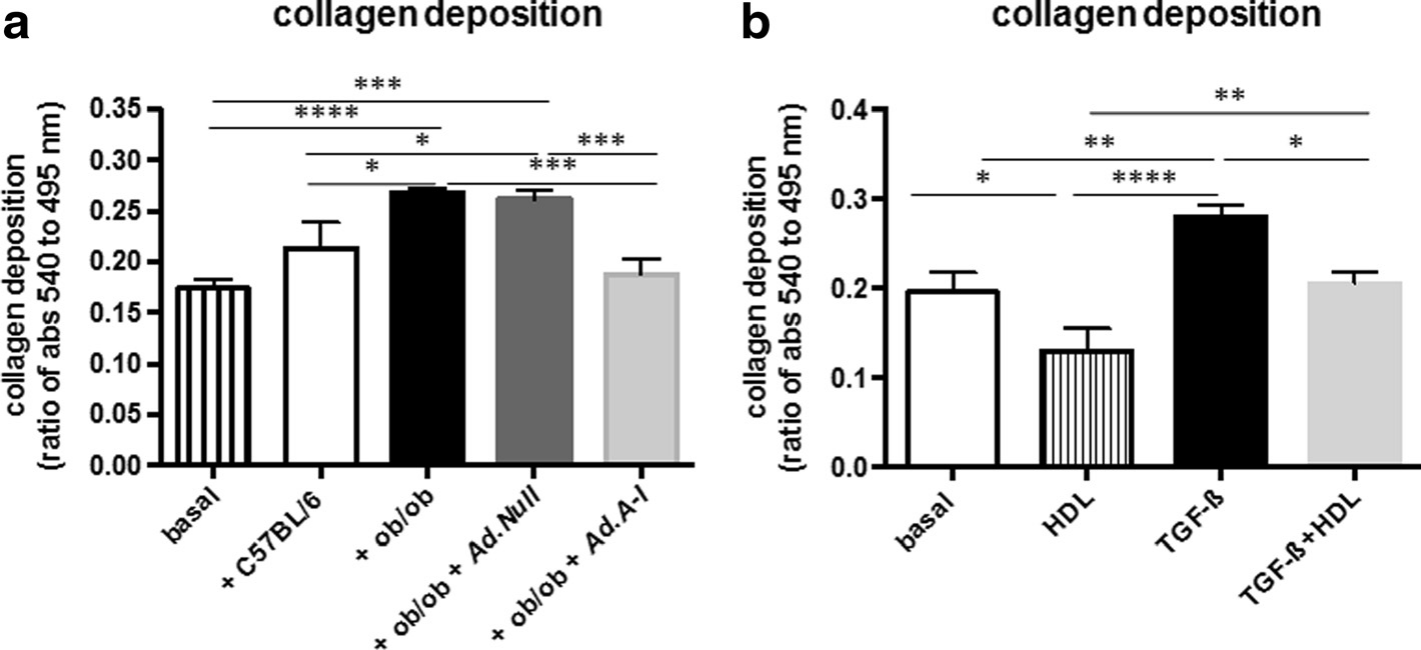
High-density lipoproteins reduce collagen deposition in murine fibroblasts via immunomodulatory and direct anti-fibrotic properties. Bar graphs represent the mean ± SEM of the ratio of the absorbance at 540 nm of Sirius Red-stained murine fibroblasts towards the absorbance at 495 nm of crystal violet-stained murine fibroblasts cultured **a**) in the absence (basal) or presence of splenocytes isolated from C57BL/6, ob/ob, ob/ob *Ad.Null* and ob/ob *Ad.A-I* mice, as indicated, at a ratio of 10 splenocytes to 1 fibroblast for 24 h, with *n =* 5-6/group and *p < 0.05, **p < 0.005, ***p < 0.0005, and ****p < 0.0001 and **b**) depict the mean ± SEM of the ratio of the absorbance at 540 nm of Sirius Red-stained murine fibroblasts towards the absorbance at 495 nm of crystal violet-stained murine fibroblasts in the presence of TGF-ß1 with/out HDL supplementation for 24 h, with the different groups as indicated: *n =* 12/control, HDL, and TGF-ß1 + HDL groups and *n =* 9/TGF-ß1 group with *p < 0.05, **p < 0.01 and ****p < 0.0001

The relevance of vascular fibrosis in obesity [3] and the anti-fibrotic effects of HDL/apo A-I [14, 27–31] further motivated us to evaluate the impact of *Ad.A-I* on the aortic mRNA expression of fibrosis markers. Ob/ob mice exhibited a 459 % (*p <* 0.0001) and 243 % (*p <* 0.0005) higher collagen I and III mRNA expression, respectively, compared to control C57BL/6 mice, whereas *AdA-I* transfer in ob/ob mice resulted in a 62 % (*p <* 0.0005) decrease of collagen I mRNA and a 66 % (*p <* 0.0005) decrease of collagen III mRNA (Fig. 6). Given the link between immune cells and fibrosis on the one hand [13] and the importance of fibrosis in vascular remodeling on the other hand [45], we next evaluated the impact of the immunomodulatory effects of *apo A-I* gene transfer on collagen production in fibroblasts upon co-culture of splenocytes isolated from the different experimental groups on fibroblasts. Co-culture of splenocytes from ob/ob and ob/ob *Ad.Null* mice with murine fibroblasts augmented collagen deposition by 54 % (*p <* 0.0001) and 50 % (*p <* 0.0005), respectively, compared to monoculture of fibroblasts. In contrast, splenocytes from ob/ob *Ad.A-I* mice and from control mice did not induce collagen content in fibroblasts upon co-culture (Fig. 7a). To evaluate whether HDL themselves reduce collagen deposition in murine fibroblasts, HDL were supplemented to fibroblasts stimulated with TGF-ß1. TGF-ß1 increased the collagen deposition in murine fibroblasts by 43 % (*p <* 0.01). In contrast, HDL reduced the TGF-ß1-induced collagen deposition to levels not different from controls (Fig. 7b).

## Discussion

The salient findings of the present study are that *apo A-I* gene transfer exerts immunomodulatory effects and decreases vascular inflammation and markers of fibrosis in ob/ob mice.

Leptin-deficient ob/ob mice are overweight [32], insulin-resistant [33], and develop type 2 diabetes mellitus over time due to deficiency of the appetite regulating hormone leptin, which has immunomodulatory properties [46]. In agreement with Xu *et al.* [47], ob/ob mice were still normoglycemic at an age of 15 weeks. However, a glucose tolerance test 4 days before sacrifice indicated a disturbance in glucose metabolism, which could not be overcome via *apo A-I* gene transfer. Furthermore, ob/ob mice exhibited hypercholesterolemia and dyslipoproteinemia (non-HDL to HDL-cholesterol ratio 1:1) in the absence of raised triglyceride levels. In agreement with *apo A-I* gene transfer studies in certain atherosclerosis models [48, 49], *Ad.A-I* transfer induced increased triglyceride levels in ob/ob mice. Under these metabolic conditions, we demonstrate that *apo A-I* gene transfer led to a downregulation of aortic TNF-α and VCAM-1 mRNA in ob/ob mice. We and others have shown that apo A-I reduces TNF-α expression [27, 50], which is an important inducer of VCAM-1 expression [44]. Downregulation of aortic VCAM-1 mRNA expression in ob/ob mice after *apo A-I* gene transfer is - on its turn - in agreement with the well-established potential of apo A-I and HDL to decrease the expression of cytokine-induced adhesion molecules [44]. This downregulated aortic VCAM-1 together with the decreased CD4 and CD8a mRNA expression in ob/ob *Ad.A-I* compared to ob/ob mice suggests a lower presence of CD4 and CD8 cells in the aorta of ob/ob *Ad.A-I* compared to ob/ob mice. Our *in vitro* findings further illustrate that HDL lower the capacity of MNC to adhere to TNF-α-activated aortic endothelial cells. This implies that HDL can decrease the adhesion of immune cells to the aortic endothelium via their endothelial-protective (lowering VCAM-1 expression) as well as immunomodulating capacity (decreasing the binding capacity of MNC to activated endothelial cells). This impaired immune cell adhesion and consequently lower presence of immune cells in the vascular wall, were expected to reduce fibrosis in the aorta of ob/ob *Ad.A-I* versus ob/ob mice, due to the lower burden of inflammation [13]. Indeed, we found that the aortic collagen I and III mRNA expression was lower in ob/ob *Ad.A-I* versus ob/ob mice, which can also be explained via a direct anti-fibrotic effect of HDL. This mechanism is corroborated by our *in vitro* finding indicating that HDL reduce the TGF-ß1-induced collagen deposition in murine fibroblasts and is further supported by our recent observation that HDL are capable of decreasing TGF-ß1-induced EndMT in aortic endothelial cells *in vitro* [14]. In this context, another explanation for the reduction in aortic collagen expression following *apo A-I* gene transfer could be the decrease in TGF-ß1-expressing splenic MNC in ob/ob *Ad.A-I* versus ob/ob mice, and subsequent lower induction of EndMT in ob/ob mice.

Evidence for the immunomodulatory capacity of *apo A-I* gene transfer in ob/ob mice follows at first from the reduction in aortic mRNA expression of the NLR proteins NOD2 and NLRP3 in ob/ob *Ad.A-I* versus ob/ob mice. NOD2 leads to NF-kB activation and subsequent expression of TNF-α and NLRP3 [51]. Its downregulation following apo A-I gene transfer can therefore partly explain the reduction in aortic TNF-α expression. Based on the involvement of NOD2 in fibrosis [11], its endothelial expression [9] and the capacity of HDL to reduce EndMT [14], we further speculate that an increase of HDL via apo A-I gene transfer can partly account for the reduction in aortic collagen I and III expression via decreasing EndMT, involving NOD2. The aortic downregulation of NLRP3, which has emerged as an important regulator of inflammation in metabolic disorders and atherosclerosis [6, 7] and is recognized for its capacity to induce collagen production in fibroblasts [12], supports the link between the decrease in vascular inflammation and fibrosis after apo A-I gene transfer.

Besides its impact on the aortic expression of NLRs, *Ad.A-I* transfer led to systemic immunomodulatory effects as indicated by a decline in the increased proliferation/activity of splenic MNC, CD4+ and CD8+ T cells in ob/ob mice. This finding is supported by Wilhelm *et al.* [24] who showed that apo A-I prevents T cell activation and proliferation in peripheral lymph nodes in mice fed an atherogenic diet. In addition, we found that *apo A-I* gene transfer reduced the percentage of splenic MNC expressing the pro-fibrotic factor TGF-ß1. Concomitantly, co-culture of splenocytes from ob/ob *Ad.A-I* mice did not induce collagen production in murine fibroblasts, in contrast to splenocytes from ob/ob and ob/ob *Ad.Null* mice, suggesting that apo A-I-raising transfer reduces the pro-fibrotic potential of splenocytes under obese conditions and their subsequent contribution to vascular fibrosis. This hypothesis, supporting the existence of a vasculosplenic axis, i.e. the migration of immune cells from the spleen to the vasculature and subsequent involvement in vascular fibrosis builds further on the existence of the cardiosplenic axis, which importance has mainly been recognized in ischemic heart disease [52]. This postulation should be taken with caution as long as the existence of a vasculosplenic axis and its relevance in vascular fibrosis is confirmed in additional experiments in ob/ob mice.

### Limitations of the study

Whereas the present study reveals systemic immunomodulatory and local (aorta) immunomodulatory/anti-fibrotic effects following apo A-I transfer in ob/ob mice, further characterization of the systemic immunomodulatory effects mice including cytokine profiles of circulating immune cells as well as the analysis of fibrosis in other vascular beds and/or adipose tissue is required to deepen our insights into the link between inflammation and fibrosis in ob/ob mice and the impact of apo A-I transfer on both parameters. Furthermore, experiments in high-fat diet-induced obesity, associated with hyperleptineamia (dysfunctional leptin), would be of value to confirm our findings.

## Conclusions

We demonstrated that apo A-I-raising transfer exerts immunomodulatory effects in ob/ob mice, including the reduction in the aortic expression of the pattern recognition receptors NOD2 and NLRP3, and the decrease in activity of splenic MNC, which are associated with a decrease in vascular inflammation and fibrosis. These findings further corroborate the immunomodulatory and vascular-protective potential of apo A-I/HDL. Furthermore, they point out that under obese conditions, modulation of the chronic low-grade activation of the innate immune system (NOD2, NLRP3) *per se*, may counteract vascular inflammation and fibrosis.

## Abbreviations

Apo, apolipoprotein; EndMT, endothelial-to-mesenchymal-transition; HDL, high-density lipoprotein; MNC, mononuclear cell; NLRP3, nucleotide-binding domain, leucine-rich-containing family, pyrin domain-containing 3; NOD2, nucleotide-binding oligomerization domain containing 2; TGF, transforming growth factor

## Additional file

Additional file 1: Figure S1. Impact of apo A-I transfer on blood glucose levels and glucose responsiveness in ob/ob mice. (TIFF 1521 kb)
