# Supplementary figures and images for: Apolipoprotein A-I gene transfer exerts immunomodulatory effects and reduces vascular inflammation and fibrosis in ob/ob mice
Source: J Inflamm (Lond). 2016 Aug 2;13:25. doi: 10.1186/s12950-016-0131-6 (PMC4969975; doi:10.1186/s12950-016-0131-6)

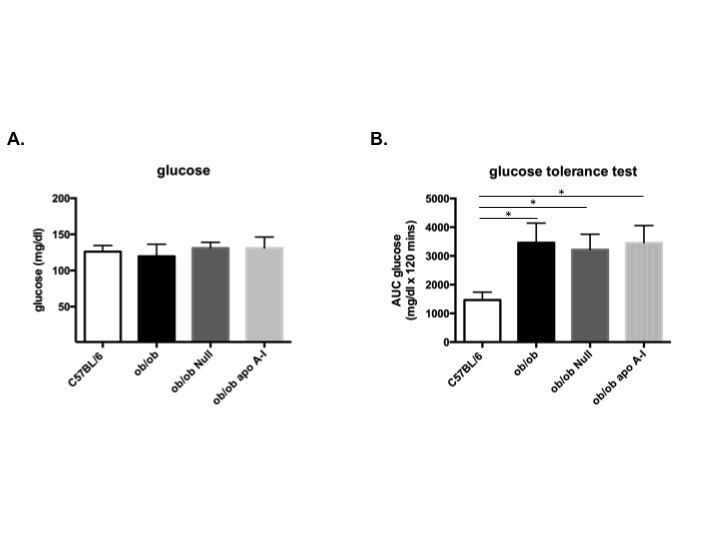

Supplement: Additional file 1: Figure S1. — Impact of apo A-I transfer on blood glucose levels and glucose responsiveness in ob/ob mice. (TIFF 1521 kb) [file 12950_2016_131_MOESM1_ESM.tiff]
